# Adenovirus-5-Vectored *P. falciparum* Vaccine Expressing CSP and AMA1. Part B: Safety, Immunogenicity and Protective Efficacy of the CSP Component

**DOI:** 10.1371/journal.pone.0025868

**Published:** 2011-10-07

**Authors:** Cindy Tamminga, Martha Sedegah, David Regis, Ilin Chuang, Judith E. Epstein, Michele Spring, Jose Mendoza-Silveiras, Shannon McGrath, Santina Maiolatesi, Sharina Reyes, Victoria Steinbeiss, Charlotte Fedders, Kathryn Smith, Brent House, Harini Ganeshan, Jennylynn Lejano, Esteban Abot, Glenna J. Banania, Renato Sayo, Fouzia Farooq, Maria Belmonte, Jittawadee Murphy, Jack Komisar, Jackie Williams, Meng Shi, Donald Brambilla, Nalini Manohar, Nancy O. Richie, Chloe Wood, Keith Limbach, Noelle B. Patterson, Joseph T. Bruder, Denise L. Doolan, C. Richter King, Carter Diggs, Lorraine Soisson, Daniel Carucci, Gail Levine, Sheetij Dutta, Michael R. Hollingdale, Christian F. Ockenhouse, Thomas L. Richie

**Affiliations:** 1 U.S. Military Malaria Vaccine Program, Naval Medical Research Center, Silver Spring, Maryland, United States of America; 2 U.S. Military Malaria Vaccine Program, Walter Reed Army Institute of Research, Silver Spring, Maryland, United States of America; 3 Clinical Research Management, Hinckley, Ohio, United States of America; 4 Henry M. Jackson Foundation for the Advancement of Military Medicine, Rockville, Maryland, United States of America; 5 RTI Rockville, Rockville, Maryland, United States of America; 6 GenVec, Inc. Gaithersburg, Maryland, United States of America; 7 USAID, Washington, D. C., United States of America; 8 Foundation for the National Institutes of Health, Bethesda, Maryland, United States of America; 9 USMMVP, Malaria Department, NMRC, Silver Spring, Maryland, United States of America; New York University School of Medicine, United States of America

## Abstract

**Background:**

A protective malaria vaccine will likely need to elicit both cell-mediated and antibody responses. As adenovirus vaccine vectors induce both these responses in humans, a Phase 1/2a clinical trial was conducted to evaluate the efficacy of an adenovirus serotype 5-vectored malaria vaccine against sporozoite challenge.

**Methodology/Principal Findings:**

NMRC-MV-Ad-PfC is an adenovirus vector encoding the *Plasmodium falciparum* 3D7 circumsporozoite protein (CSP). It is one component of a two-component vaccine NMRC-M3V-Ad-PfCA consisting of one adenovector encoding CSP and one encoding apical membrane antigen-1 (AMA1) that was evaluated for safety and immunogenicity in an earlier study (see companion paper, Sedegah et al). Fourteen Ad5 seropositive or negative adults received two doses of NMRC-MV-Ad-PfC sixteen weeks apart, at 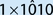 particle units per dose. The vaccine was safe and well tolerated. All volunteers developed positive ELISpot responses by 28 days after the first immunization (geometric mean 272 spot forming cells/million[sfc/m]) that declined during the following 16 weeks and increased after the second dose to levels that in most cases were less than the initial peak (geometric mean 119 sfc/m). CD8+ predominated over CD4+ responses, as in the first clinical trial. Antibody responses were poor and like ELISpot responses increased after the second immunization but did not exceed the initial peak. Pre-existing neutralizing antibodies (NAb) to Ad5 did not affect the immunogenicity of the first dose, but the fold increase in NAb induced by the first dose was significantly associated with poorer antibody responses after the second dose, while ELISpot responses remained unaffected. When challenged by the bite of *P. falciparum*-infected mosquitoes, two of 11 volunteers showed a delay in the time to patency compared to infectivity controls, but no volunteers were sterilely protected.

**Significance:**

The NMRC-MV-Ad-PfC vaccine expressing CSP was safe and well tolerated given as two doses, but did not provide sterile protection.

**Trial Registration:**

ClinicalTrials.gov NCT00392015

## Introduction

*P. falciparum* malaria causes 863,000 deaths and approximately 243 million cases annually and is a major infectious threat to non-immune travelers to malaria-endemic areas[1]. Increasing drug and insecticide resistance highlight the importance of developing an effective malaria vaccine [2]. Sterile protective immunity against malaria can be induced in animals or humans with radiation-attenuated sporozoites, delivered by mosquito bite, that invade hepatocytes, develop partially but are unable to transform into blood stage parasites [3], [4], [5]. Protection appears to be species- but not strain-specific, is sustained for at least nine months, and is probably dependent on cell-mediated immunity (CMI) against infected hepatocytes and antibodies against sporozoites [6], [7], [8], [9], [10]. This model of protective immunity provides a rationale for developing a vaccine inducing CMI targeting pre-erythrocytic stages. On the other hand, naturally-acquired immunity to malaria in humans appears to be dependent primarily on antibody responses against blood stage antigens[11], [12], with T cell responses contributing[13], [14], providing a rationale for including blood stage antigens in a multi-stage vaccine.

NMRC-M3V-Ad-PfCA is a multistage adenovirus serotype 5 (Ad5)-vectored *P. falciparum* (3D7 strain) malaria vaccine containing two adenovectors mixed together for intramuscular delivery, one encoding the circumsporozoite protein (CSP) and the second the apical membrane antigen-1 (AMA1). The vaccine is designed to reproduce the protective immune responses afforded by the irradiated sporozoite vaccine (pre-erythrocytic stage immunity) and by natural exposure (blood stage immunity). CSP, the major surface protein of sporozoites, was chosen based on the ability of the RTS,S vaccine, comprised of recombinant CSP, to elicit protection in adults [15], children and infants [16] and due to CSP's contribution to the immunity induced by irradiated sporozoites[17], [18], [19]. AMA1[20], expressed by sporozoites, liver stages and merozoites[21], [22], [23], [24], was selected because naturally-acquired anti-AMA1 antibodies and proliferative responses are associated with protection in endemic areas [25], [26], [27], recombinant AMA1 protein is protective in non-human primates[28], and has proven safe and immunogenic in Phase 1 studies in humans [29], [30], [31], [32], [33], [34].

Adenovirus-based vaccines may be better suited for inducing immune responses which attack developing liver stage parasites than recombinant proteins, based on their ability to induce antigen-specific CD8+ T cells when administered either alone or in heterologous regimens [35], [36], [37], [38], [39], [40], [41], [42], [43], [44], [45], [46]. In contrast the RTS,S vaccine is thought to protect primarily via antibody and CD4+ T cell responses to CSP, effectively targeting the sporozoite[16], [47], [48], [49], and does not appear to elicit significant CD8+ T cell responses [50], [51]. Recombinant attenuated adenoviruses induce protection against malaria in animal models[52], including adenovectors encoding CSP [53], [54]. For this reason, it seemed reasonable to determine whether an adenovirus *P. falciparum* CSP vaccine alone would also be protective in humans, before combining with AMA1, and before combining with a heterologous vaccine in a prime-boost strategy.

In an initial clinical study, the safety of the parent, two-component vaccine, NMRC-M3V-Ad-PfCA, was evaluated at two doses in Ad5 seronegative volunteers (see companion paper Sedegah et al, Groups 1 and 2). CD8+ T cell responses were significantly stronger at the lower dose studied. In this second clinical study (Group 3), the CSP component of the vaccine, NMRC-MV-Ad-PfC, was evaluated at the lower dose for safety, immunogenicity and protection against experimental sporozoite challenge, enrolling both Ad5 seronegative and seropositive volunteers. The trial demonstrated that the vaccine was safe and well tolerated, but the second dose did not improve immunogenicity over the first dose, and the vaccine did not sterilely protect any volunteers.

## Methods

The protocol for this trial and supporting CONSORT checklist are available as supporting information; see Checklist S1 and Protocol S1.

### Ethics

The Trial Protocol for the clinical trial presented in this manuscript was approved by the National Naval Medical Center (NNMC), Naval Medical Research Center (NMRC) and Walter Reed Army Institute of Research Institutional Review Boards, in compliance with all applicable federal regulations governing the protection of human subjects. All study subjects gave written informed consent. This study was conducted according to all Federal Regulations regarding the protection of human participants in research including The Nuremberg Code, The Belmont Report, 32 CFR 219 (The Common Rule) and all pertinent standards for the responsible conduct of research set by the Department of Defense, the Department of the Navy, the Department of the Army, the Navy Bureau of Medicine and Surgery, the NNMC, the NMRC and the US Army Medical Research and Materiel Command. NMRC holds a Department of Defense/Department of the Navy Federal Wide Assurance for human subject protections, and a Federal Wide Assurance from the Office for Human Research Protections for cooperation with the Department of Health and Human Services (FWA 0152). All NMRC personnel were certified as having completed mandatory human research ethics education curricula and training under the direction of the NMRC Office of Research Administration and Human Subjects Protections Program. The trial was performed under US Food and Drug Investigational New Drug Application BB-IND-13003.

### Participants

Healthy malaria-naïve civilian and military adult men and women, age 18–50 years, were recruited for this study that was conducted at the NMRC Clinical Trials Center in Bethesda, Maryland. After obtaining informed consent, volunteers were screened using inclusion and exclusion criteria similar to those described in the companion paper (Sedegah et al). However, the definition of “malaria naïve” was refined for Part B of the study; an exclusion criteria was added prior to the challenge to exclude volunteers determined to have a greater than 10% five year risk for a cardiovascular event based on the non-invasive cardiac assessment tool outlined by Gaziano[55]. The Trial Protocol is provided as part of the supplementary material.

### Interventions

The vaccine, an adenovirus-derived construct expressing *P. falciparum* strain 3D7 CSP (NMRC-MV-Ad-PfC), is described in Sedegah et al (Groups 1 and 2). Immunization of Group 3 was initiated in July 2008, five months after the final high dose immunization with the parent vaccine. Fifteen volunteers received the first dose and 14 the second dose. Each volunteer received 1×10^10^ particle units (pu)/mL. The dose of the CSP-encoding construct was the same as the dose of CSP-encoding construct in Group 1 in Sedegah et al (Table 1).

**Table 1:** Vaccine constructs, doses, and times of immunization.

Vaccine	Trial	Group (n)	Particle units (pu) per dose			# doses	Total dose (pu)
			CSP	AMA1	Total		
NMRC-M3V-Ad-PfCA (CSP & AMA1 mixed)	1	1 (6)	1×10^10^	1×10^10^	2×10^10^	1	2×10^10^
		2 (6)	5×10^10^	5×10^10^	1×10^11^	1	1×10^11^
NMRC-MV-Ad-PfC (CSP only)	2	3 (15)	1×10^10^	0	1×10^10^	2	2×10^10^

**Trial 1**: Dose escalation study: Group 1 was immunized with a single dose, a safety review was conducted, then Group 2 was immunized with a single five-fold higher dose. Results are presented in the companion publication, Sedegah et al.

**Trial 2**: Challenge study. Group 3 was immunized twice using the Group 1 dose of CSP, with 15 weeks between doses. Fifteen volunteers received the first dose, 14 volunteers the second; data for all 29 immunizations are included in the safety analysis. Twelve volunteers underwent malaria challenge 21 days following the second immunization; one of the 12 was later excluded from the analysis of immunogenicity and protection due to the discovery of prior participation in another malaria challenge trial.

### Objectives

The first objective was to assess the safety and tolerability of the NMRC-MV-Ad-PfC vaccine. The second objective was to assess the immunogenicity of the vaccine, including the effects of Ad5 seropositivity. The third objective was to assess the protective efficacy against sporozoite challenge, enabling historical comparison with the protection afforded by RTS,S.

### Outcomes

The methods used to evaluate safety, tolerability and reactogenicity were very similar to those described in the companion paper, Sedegah et al. For this part of the study, safety assessments were recorded by direct observation and questioning on days 1, 2, 3, 5, 7, 14 and 28. The final study visit was approximately 1 year after the second immunization. Local, systemic, and laboratory AEs were graded using severity scales detailed in the Trial Protocol (Sedegah et al, supplementary material). Telephone or email follow-up for all study subjects is to extend for a total of five years per FDA request.

### Sample size

The sample size was based on many years of experience conducting malaria vaccine trials at NMRC and WRAIR and was designed to demonstrate the vaccine's safety and tolerability profile in a small number of volunteers to provide evidence that the frequency of serious or severe vaccine-related AEs was sufficiently low to continue testing in larger numbers of volunteers. If none of the targeted twelve immunized volunteers experienced a serious or severe vaccine-related AE, the following predictions could be made regarding vaccine safety: there was a 46% level of confidence that the true rate of severe or serious vaccine-related AEs in the general population would be less than 5%; alternatively, there was a 72% level of confidence that the true rate of these events in the general population would be less than 10%; or, as a third example, there was a 93% level of confidence that the true rate would be less than 20%. These figures were determined using the exact binomial method (1-p) n = 1-c where p is the probability that a subject has an event, n is the total number of subjects and c is the level of confidence. In addition the sample size was powered to detect a two day mean delay in patency in the immunized group compared to the infectivity controls (80% power, α = 0.05, one-sided) [15].

### Immunological endpoints

Immunological measurements were performed pre-immunization, 1 month and 15 weeks after the first immunization, 19 days after the second immunization, and 3 weeks after challenge.

#### *Ex vivo* Enzyme-Linked Immunospot (ELISpot) IFN-γ activity

This was performed as described in Sedegah et al. Stimulants were pools of 15 amino acid (aa) synthetic peptides overlapping by 11 aa (Chiron Technologies, Clayton, Victoria, Australia) covering full length CSP. These were combined into 9 CSP pools (Cp1-Cp9) containing 3-12 peptides per pool (Table 2), with pools used individually as stimulants for fresh PBMCs. Each pool (except Cp2) contained previously identified CSP T epitopes[56], [57], [58]

**Table 2:** CSP peptides used in ELISpot and ICS assays.

Pool	CSP aa	# Pep*	Class	HLA restriction	Residues	Sequence**
**1** +	1–39	7	I	A2.1 supertype	1–10	MMRKLAILSV
			I	A2 supertype	7–16	ILSVSSFLFV
			II	DR (A2.1 and A2 supertype)	1–20	MMRKLAILSVSSFLFVEALF
**2** +	29–71	8				
3	61–107	9	I	B8	81–89	KLRKPKHKK
4	97–283	12	II	DR	105–116	NANPNVDPNANP
5	273–319	9	II	DR (B7)	281–300	QGHNMPNDPNRNVDENANAN
			I	B7	86–94	MPNDPNRNV
**6** +	309–331	3	I	A1	310–319	EPSDKHIKEY
			I	A2.1	319–327	YLNKIQNSL
			II	DR (A2.1)	316–335	IKEYLNKIQNSLSTEWSPCS
7	321–335	6	II	Th2r	326–343	SLSTEWSPCSVTCGNGIQ
8	345–367	3	II	B35-Th3r	346–365	IKPGSANKPKDELDYANDIE
**9** +	357–397	8	II	DR	363–383	DIEKKICKMEKCSSVFNVVNS
			II	DR (A2 supertype)	375–397	SSVFNVVNSSIGLIMVLSFLFLN
			I	A2 supertype	386–394	GLIMVLSFL

PfCSP peptide sequences and residue numbers were based on those of the *P. falciparum* clone 3D7 (GenBank no. X15363). Previously identified Class I and II CSP epitopes were distributed among peptide pools, except pool 2.

**Previously identified T epitopes.

*Number of 15mer peptides in each pool.

**As described in references 55–57.

+ Peptide pools used in ICS assays.

#### Intracellular cytokine staining (ICS)

For each volunteer, matched pre- and post-vaccination samples were tested simultaneously using frozen PBMCs. ICS was performed as published previously [38] and is described in Sedegah et al.

#### Enzyme-Linked Immunosorbent Assay (ELISA)

ELISA was used to measure total IgG antibody titers against the *P. falciparum* CSP central repeat region using a hexameric synthetic peptide (NANP)_6_ as the capture antigen as described in Sedegah et al. A positive antibody response required that the means of the post-immunization antibody responses (endpoint titers) differed significantly from pre-immunization responses (*P*<0.05) using the paired Student's two-tailed *t*-test [59].

#### Immunofluorescent Antibody Assay (IFA)

*P. falciparum* sporozoite-specific antibodies were assayed by immunofluorescent staining of air-dried *P. falciparum* sporozoites as described previously[60].

#### Adenovirus serotype 5 neutralization assay

This test was performed under contract by the NIAID Vaccine Immunogenicity T-cell and Antibody Laboratory (NVITAL), Gaithersburg, MD. Sera that had been heat inactivated for 60 minutes at 56°C were serially diluted in a final sample volume of 50 µL D10 (Dulbecco's modified Eagle's medium supplemented with 10% heat inactivated fetal bovine serum, penicillin 100 U/mL, and streptomycin 100 µg/mL). To each well 50 µL of the optimized dilution of rAd5-luciferase was added, followed by addition of 1×10^4^ A549 cells (human lung carcinoma)/well in 100 µL D10. The plates were incubated at 37°C in 10% CO_2_ for 24 hours. After centrifugation to pellet the cells they were resuspended in 100 µL Glo Lysis Buffer. The cell suspension was transferred to a Black and White Isoplate (Perkin Elmer) and 100 µL Steady-Glo Luciferase Assay System Reagent (Promega) added per well. Following incubation at room temperature for 15-minutes luminescence was measured on a luminometer. The 90% inhibition serum titer was determined to be that serum dilution which could be interpolated to have 10% of the maximum luciferase activity as determined by the assay run without the presence of a serum sample.

### Efficacy

Protective efficacy was assessed by conducting a homologous 3D7 strain sporozoite challenge 3 weeks after the second NMRC-MV-Ad-PfC immunization. Twelve vaccinated and 6 unvaccinated volunteers (infectivity controls) underwent a standardized challenge as previously described [61]. Starting on day 7 after challenge, volunteers were housed at the Navy Lodge on the NNMC campus in Bethesda, MD. Each volunteer was monitored for the onset of signs and symptoms of malaria and by daily Giemsa-stained thick blood films with positive films confirmed by a second reader. The identity of immunized and non-immunized volunteers was known to the clinical trial staff but not to the microscopists reading the malaria smears. Symptomatic, undiagnosed volunteers had additional smears performed at the discretion of the study doctor, not to exceed one smear every 8–12 hours. Volunteers who developed malaria (as defined by a positive blood smear) were treated with a standard oral dose of chloroquine: total 2500 mg base given in divided doses: 1000 mg initially followed by 500 mg at 6, 24 and 48 hours under direct observation.

#### Microscopy

10 µl of blood was smeared onto each of two 1 cm×2 cm rectangles on each slide. The slides were dried on a 37°C heat block for 5–10 minutes. Slides were stained with fresh Giemsa stain (4% solution of stain in phosphate buffered water) for 45–60 minutes, rinsed with water and allowed to dry. The slides were viewed under oil immersion at a total of 1000x magnification. For asymptomatic individuals, 360 fields were counted in five vertical passes. For symptomatic individuals, 1080 fields were counted in approximately 15 vertical passes. A volunteer was determined to be parasitemic when at least two parasites were found and confirmed by an expert microscopist. The physician on duty was informed by the microscopist of the volunteer's status and personally observed the confirmed parasites. Parasitemia was calculated from the number of parasites observed in five passes (0.45 mm^3^) multiplied by 2.22 to give parasites/µl. The expert microscopist had extensive experience in malaria microscopy and all microscopists passed a proficiency test on slide parasite detection.

### Statistical methods

ELISpot activities were expressed as sfc/m and as the geometric means of each group. ELISA and IFA activities were expressed as titers and as the medians of each group. Variation in ELISpot counts, ELISA measurements, IFA measurements and flow cytometry data over time within groups was examined using repeated measures models. ELISpot, ELISA and IFA, but not flow cytometry, data were transformed to logarithms for analysis to stabilize variances. Linear contrasts were employed to compare specific time points if variation over time was detected. Repeated measures models were also employed to compare trends over time among Groups 1, 2 and 3. The initial model in each analysis included the group, a categorical variable for time, and the group-time interaction as predictors. The interaction provided a test for differences among groups in changes over time. The interaction was dropped if it was not statistically significant. Tests for variation over time and differences between groups were based on the reduced model. A statistically significant interaction was followed by more detailed analysis. If the initial analysis involved all three groups, then the analysis was repeated for each of the three pairs of groups to elucidate the reasons for the interaction. The same approaches were used to examine trends in log-transformed Ad5 neutralizing antibodies within each of three subgroups and to compare trends among the subgroups, with the subgroups defined by baseline neutralizing antibody titer (<12, ≥12 but ≤500, >500).

Time to parasitemia in the vaccinated subjects and controls was compared using Kaplan-Meier survival curves with a log-rank statistic. For each vaccinated volunteer, delay to parasitemia was defined as significantly positive if the time to parasitemia was 2 standard deviations greater than the geometric mean of the time to patency of the controls[49]. Rank correlations were employed to examine the relationships between Ad5 neutralizing antibodies and measures of immunogenicity. The number of AEs reported per subject was compared between groups using the Wilcoxon two sample test to compare Group 1, Group 2 (Sedegah et al) and Group 3, or the three subgroups defined by Ad5 neutralizing antibody titer (≤12, 12–500, >500).

## Results

### Participant flow

**Figure 1:**
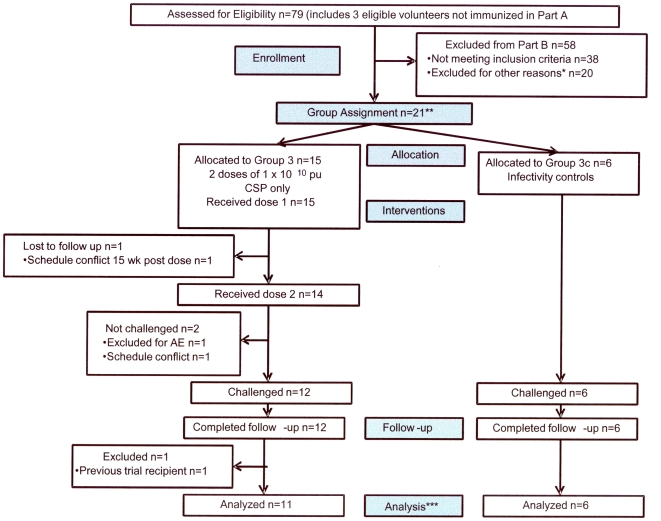
Flow diagram of volunteers (Group 3). * Reasons for exclusion: Moved out of area (3), deployed (2), lost to follow up (2), opted out (1), eligible but preferred waiting for follow-on study (12). ** The first 15 volunteers were allocated to the vaccine group and the final six volunteers to infectivity controls. *** 11 volunteers analyzed for immunogenicity and protection; 15 analyzed for safety of dose 1, 14 for safety of dose 2.

The participant flow is shown in Figure 1. Recruitment for Group 3 took place between February and June 2008 for the immunization phase and between July and November 2008 for the challenge phase (enrolment of six infectivity controls). Seventy-nine healthy, malaria-naïve, civilian and military adult men and women were recruited and screened for eligibility in a manner similar to that used for Groups 1 and 2 except volunteers with pre-existing NAb to Ad5 were included. Twenty-one volunteers who met all screening criteria participated as vaccine recipients (n = 15) or as infectivity controls (n = 6). One volunteer withdrew prior to the second immunization due to schedule conflicts, another volunteer withdrew prior to the challenge due to schedule conflicts, and a third volunteer was withheld from the challenge due to safety concerns related to a Grade 3 headache experienced following the second immunization (see below). Therefore 15 volunteers received the first immunization, 14 the second, and 12 participated in the malaria sporozoite challenge. Of these 12, one was excluded after challenge from the immunogenicity and efficacy analyses but not safety analyses when it was discovered that he had been a participant in a previous malaria vaccine study and therefore may have possessed a degree of immunity to malaria. In total, the NMRC-MV-Ad-PfC vaccine was administered as 29 doses to 15 volunteers at 1×10^10^ pu. The demographic make-up of the participant volunteers is shown in Table 3. The vaccine groups and infectivity controls were similar in age and ethnicity.

**Table 3:** Demographics of volunteers.

	Immunized N = 15	Controls* N = 6
Male #	11	3
Female #	4	3
Age range 18–20 years	0	0
Age range 21–30 years	4	2
Age range 31–40 years	6	1
Age range 41–50 years	5	3
Overall age range years	21–50	26–48
Median age years	36.5	40.0
Mean age years	35.9	38.0
Caucasian #	4	5
African American #	8	1
Hispanic #	1	0
Asian #	2	0
Individual Ad5 NAb titers	<12,<12, <12<12, 82, 114, 157, 615, 5068>8748,>8748**	Not applicable

*Infectivity controls.

**NAb = neutralizing antibody titers for Ad5 (provided only for the 11 volunteers included in the immunogenicity and efficacy analysis).

### Local and systemic adverse events

Solicited post-immunization AEs recorded in each 14-day follow-up period are shown in Table 4. The only reported local AEs were pain and/or tenderness at the injection site, which occurred in ten of 15 (67%) of the volunteers following the first immunization and in 12 of 14 (86%) of the volunteers following the second immunization. The majority of these were of moderate severity. No adenopathy was recorded, although ipsilateral axillary tenderness was noted in one volunteer following the first immunization. There were no Grade 3 local AEs following either immunization.

**Table 4:** Number (percent) of volunteers experiencing solicited local and systemic adverse (definitely, probably or possibly related to immunization).

Sign or Symptom	1^st^ Imm (n = 15)		2^nd^ Immun (n = 14)	
	All	Gr 3	All	Gr 3
LOCAL				
Pain/tenderness	10(67)	0	12 (86)	0
Erythema	0	0	0	0
Induration/swelling	0	0	0	0
Warmth	Ns	-	0	0
Hives	Ns	-	0	0
Lymphadenopathy	0	0	0	0
Limited arm motion	Ns	-	0	0
Axillary tenderness	1 (7)	0	0	0
**MEAN** † **LOCAL**	**0.7**	**0**	**0.9**	**0**
**SYSTEMIC**				
Fever (objective) (((O((objective)(objective)	0	0	0	0
Fever (subjective)	1 (7)	0	0	0
Headache	1(7)	0	2(14)	1(7)
Malaise	2(13)	0	1(7)	0
Fatigue	1(7)	0	2(14)	0
Myalgia	2(13)	0	1(7)	0
Arthralgia	0	0	0	0
Nausea/vomiting	0	0	2(14)	0
Diarrhea	1(7)	0	1(7)	0
Pink eye/conjunctivitis D	0	0	1 (7)	0
Nasal congestion	1 (7)	0	0	0
Dysuria	0	0	0	0
Pharyngitis	ns*	-	1 (7)	0
Eye pain/irritation	ns*	-	1 (7)	0
Dizziness	ns*	-	1 (7)	0
Chills	ns*	-	1(7)	0
**MEAN** † **SYSTEMIC**	**0.6**	**0**	**1.0**	**0.1**

Solicited adverse events were recorded for 14 days after each immunization. Gr = severity grade.

† Mean number per volunteer per immunization.

*Not solicited (ns) after first immunization, but added to the list of solicited symptoms after second immunization. The following were also added for the second immunization with negative responses by all volunteers: pruritis, rash, cough, coryza, and urinary frequency.

The most common systemic vaccine-related AEs following the first immunization were malaise and myalgia, each occurring in two of 15 (13%) volunteers, and following the second immunization, fatigue, headache, nausea and/or vomiting, each occurring in two of 14 (14%) volunteers. Systemic events following both the first and second immunization were mostly of mild severity.

One Grade 3 AE was reported, a headache that began approximately 8 hours following receipt of the second immunization in a volunteer with a history of migraine headaches. The headache was associated with photosensitivity and sound sensitivity, typical for this volunteer's migraine headache pattern. The volunteer also experienced 3 episodes of moderate (Grade 2) chills during the headache, occurring 12–13 hours following the second immunization. The chills were not a typical component of this volunteer's migraine pattern. The volunteer reported prompt relief of the headache and associated symptoms on taking prescribed migraine medication. The event was deemed possibly related to the vaccine and the volunteer was excluded from the malaria sporozoite challenge.

Because of the theoretical, even if remote, possibility of reversion of the vaccine to replication competence, attention was paid to symptoms that might have reflected adenovirus infection. Several volunteers experienced clinical syndromes of mild or moderate severity consistent with adenovirus infection, including conjunctivitis, enteritis, nasal congestion, pharyngitis and upper or lower respiratory infections (see also Table S1: Unsolicited Adverse Events), but the timing relative to immunization was variable suggesting that these were probably not related to immunization. The rate of occurrence of these miscellaneous clinical syndromes appears consistent with background infections circulating in the community during the periods of immunization.

### Comparing Groups 1, 2 and 3

**Figure 2:**
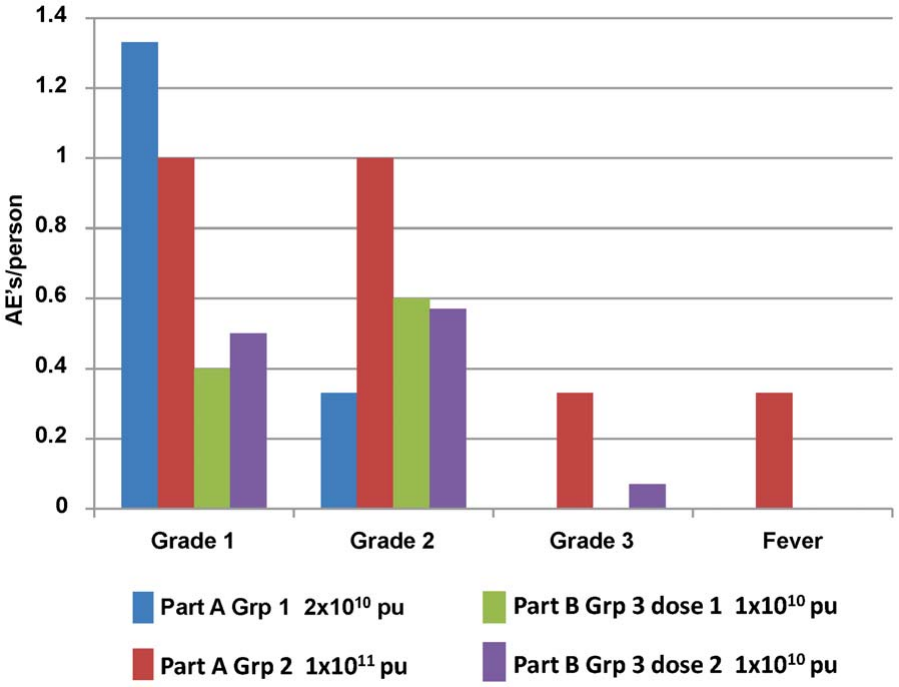
Frequency of Selected Adverse Events Recorded from Day of Immunization through 14 days post-immunization for Groups 1, 2 and 3. The frequencies of pain, malaise, headache and myalgia (combined) by severity grade, and, separately, of objective fever (one grade 1 and one grade 2) are shown for each vaccine group.

To more closely investigate whether there was a relationship between dose of the adenovector and AE severity, we examined the differences among the three groups of volunteers receiving the vaccine (see Sedegah et al). As derived from Table 4 the frequencies per volunteer of four common AEs (injection site pain or tenderness, malaise, headache and myalgia) and of fever were compared (Figure 2). On average, subjects in high dose Group 2 (Sedegah et al) reported the greatest number of AE's (Grade 1: 1.0/volunteer, Grade 2: 1.0/volunteer, Grade 3: 0.33/volunteer), while those after the first immunization in Group 3 (this study) reported the fewest (Grade 1: 0.4/volunteer, Grade 2: 0.6/volunteer, Grade 3: 0.00/volunteer). Although the differences were not statistically significant using the Wilcoxon two sample test after the first (p = 0.08) or second (p = 0.10) immunizations in Group 3, the trends in these differences among groups were consistent with the varying doses of adenovector received: Group 2 received 5-fold more per dose than Group 1, and 10-fold more than Group 3.

### Effect of pre-existing anti-Ad5 antibodies and adverse events

Although pre-existing anti-Ad5 antibodies have been reported to reduce the incidence of AEs in some clinical trials of adenovirus vectored vaccines[62], there was no association between pre-existing anti-Ad5 antibody titer and the sum of local and systemic AEs (Pearson Correlation p = 0.70, 2-sided). The second immunization was excluded from the analysis because all subjects became seropositive after the first immunization.

### Unsolicited adverse events

Volunteers were questioned for unsolicited symptoms for 28 days following vaccine administration by being asked “are you experiencing any additional symptoms?” Their unsolicited AEs are listed in Table S1. The unsolicited AEs considered definitely or probably related to immunization were: burning (n = 1), numbness (n = 1) or bruising (n = 2) at the injection site.

### Laboratory adverse events

**Figure 3:**
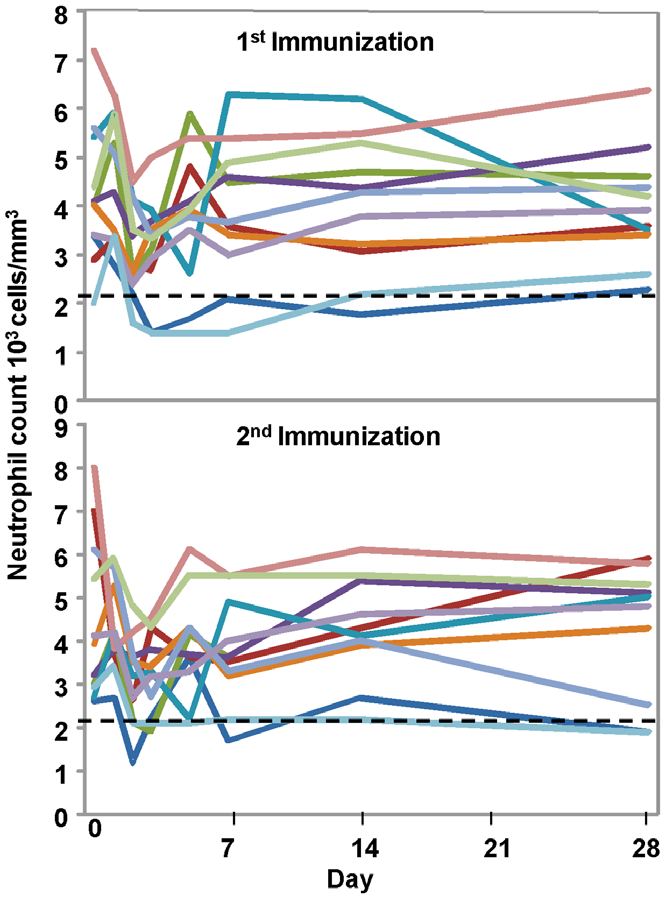
Neutrophil Kinetics Group 3. A fall in neutrophil count was observed in most volunteers at 2–3 days post-immunization with return to normal or near normal levels by day 7 post-immunization after the 1st and 2nd immunization. Day 0 for the 2nd immunization is 15 weeks after the 1st immunization. Dotted line indicates normal levels.

In response to the transient falls in white blood count seen in Groups 1 and 2, the Trial Protocol for Group 3 was amended to include more frequent collection of safety labs during the first week post immunizations namely days 0, 1, 2, 3, 5 and 7. As an unrelated change, the chemistry panel was reduced in scope, to only include creatinine, aspartate aminotransferase (AST) and alanine aminotransferase (ALT). As in Groups 1 and 2, an overall decrease in WBC, neutrophil and lymphocyte counts occurred in the majority of the volunteers within the first 3 days after receiving the vaccine (Figure 3). Within 14 days after the first immunization, Grade 1 neutropenia was recorded in one of 15 volunteers and Grade 2 in one of 15 volunteers; within 14 days after the second immunization, Grade 1 neutropenia was recorded in two of 14 volunteers and Grade 2 in two of 14 volunteers.

In general the decrease in lymphocytes occurred earlier than the decrease in neutrophils (day 1 compared to day 2 or 3) (data not shown). After immunization one, three volunteers had elevated AST and/or ALT; all were Grade 1 except for a Grade 2 ALT in one volunteer. After immunization two, four volunteers had elevated AST and/or ALT; all were Grade 1. A few volunteers had mild abnormalities on urinalysis. No other laboratory trends were noted.

### Immunogenicity

Cell mediated and antibody responses were measured pre-immunization, 1 month and 15 weeks after the 1^st^ immunization, 19 days after the 2^nd^ immunization (2 days prior to challenge) and 21 days after challenge.

#### T cell responses to CSP by ex vivo IFN-γ ELISpot Assay

**Figure 4:**
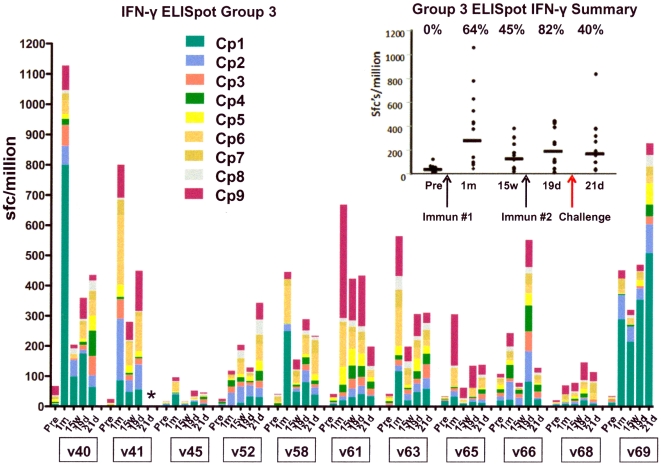
ELISpot IFN-γ CSP. The ELISpot activity of each volunteer at pre-immunization, 1 month and 15 weeks after the first immunization, 19 days after the second immunization, and 21 days after challenge (* this last data point not available for volunteer 41), as stacked, color-coded peptide pool-specific responses at each time point. The insert shows the values of the sum of pool-specific responses for each volunteer at each time point. The horizontal bar indicates the geometric mean of the group, the arrows indicate the first and second immunizations and challenge, and the percent of volunteers who were positive with at least one CSP pool at each time point is written at the top of the insert.

All 11 volunteers developed increasing responses to CSP following the first immunization, as measured by fresh, *ex vivo* (36 hour stimulation) ELISpot assay (seven of 11 meeting criteria for positive–see Methods, Sedegah et al), with the responses of nine peaking at 4 weeks and then drifting down by 15 weeks, while two (v52 and v68) had higher responses at 15 weeks than at 4 weeks (Figure 4). Geometric mean responses at 4 weeks were 323 sfc/m, range 71–1127 sfc/m, and decreased by 15 weeks to 152 sfc/m, range 63–320 sfc/m. All volunteers but one (v52) again developed increasing responses to CSP by ELISpot assay following the second immunization (nine of 11 meeting criteria for positive), but in seven of 11 cases the peak response following the second immunization was less than the peak observed following the first immunization. Geometric means at 19 days after the second immunization were 247 sfc/m, range 127–552 sfc/m. When the group was examined as a whole, there was a statistically significant increase in ELISpot responses with each immunization (p<0.0001 for the first and p = 0.02 for the second immunization, ANOVA), but there was no difference between the peak responses following the two immunizations (p = 0.22, ANOVA) (Figure 4 insert). V45 was the weakest responder by ELISpot (one of two volunteers not meeting criteria for a positive response) and also by ELISA and IFA (see below). Volunteers responded to a variety of peptide pools, with Cp1, Cp2, Cp6 and Cp9 inducing the strongest recall responses overall, as was the case for Groups 1 and 2. At least three volunteers showed boosting of ELISpot responses following challenge (v40, v52 and v69) but others did not, and overall group responses were unchanged following challenge.

#### Total-IFN-γ (T- IFN-γ) responses to CSP peptides by intracellular cytokine staining (ICS)

**Figure 5:**
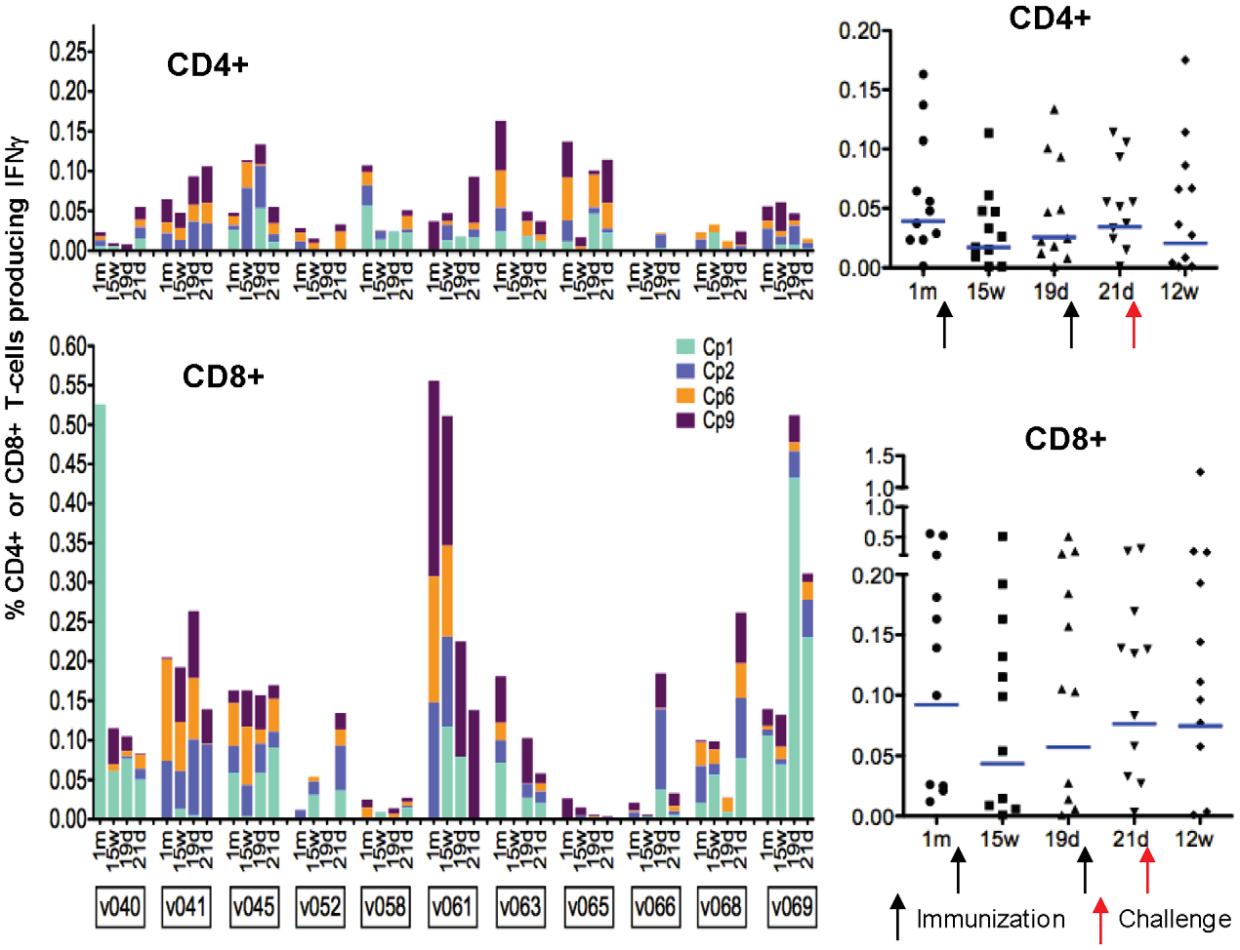
ICS CD4+ and CD8+ T cell IFN-γ CSP responses. Percent CD4+ and CD8+ T cells secreting IFN-γ in response to CSP for each volunteer at each time point, color-coded by peptide pool. The percents at each time point are plotted for each volunteer along with the geometric means for the group (horizontal bars). Responses prior to immunization are subtracted from all data in order to show only vaccine-induced responses.

Multi-parameter flow cytometry ICS assays were conducted on frozen PBMC at the same time points as fresh ELISpot assays using the four CSP peptide pools most strongly stimulating recall responses in ELISpot assays (Cp1, Cp2, Cp6 and Cp9). Results are expressed as percentage gated CD4+ or CD8+ T cells secreting IFN-γ after subtraction of pre-immunization values (Figure 5). As with Groups 1 and 2 ELISpot activities, overall CD8+ T cell IFN-γ responses were significantly stronger than CD4+ T cell IFN-γ responses (p = 0.026), despite the fact that two volunteers (v058 and v065) showed stronger CD4+ T cell responses. Overall, there were numerically more IFN-γ-secreting CD8+ T cells that CD4+ T cells even after correcting for the larger number of gated CD4+ T cells (data not shown).

As with ELISpot, the ICS responses of most volunteers peaked four weeks after the first immunization (geometric means CD8+ 0.09% range 0.012–0.556%, CD4+0.039% range 0.00141–0.137%), fell during the ensuing 11 weeks, and were then boosted by the second immunization (geometric means CD8+ 0.046% range 0.001–0.512%, CD4+ 0.021% range 0.0081–0.1338%) but generally not to the levels achieved after the first immunization; however, a few volunteers' responses were higher after the second immunization compared with after the first. Overall geometric mean IFN-γ responses were not statistically different after the second immunization compared with after the first immunization for CD4+ or CD8+ T cells (Figure 5). Responses following challenge varied among volunteers, some rising and some falling, and overall there was no statistically significant difference from values prior to challenge.

#### Multifunctional T cell responses to CSP

In addition to IFN-γ responses, TNF-α and IL-2 responses were measured, including the frequencies of CD4 and CD8 T cells producing only these cytokines or these cytokines in combination with others (multifunctional T cells). The frequencies of multifunctional cells, defined as those producing two or more cytokines, at 1 m are shown in Figure S1. As was seen in Groups 1 and 2, the frequencies of CD8+ and CD4+ multifunctional T cells were similar after the first immunization (geometric means CD8+ 0.014% range 0.001–0.087, CD4+ 0.015% range 0.001−0.0631), and this was also true after the second immunization (geometric means CD8+ 0.008% range 0.001−0.196, CD4+ 0.011% range 0.001−0.089). Pool-specific multifunctional responses were examined and the geomeans showed similar magnitudes for CD4+ and CD8+ T cells (p = 0.8), with a great deal of variation among individual vaccine recipients especially for multifunctional CD8+ T cells (Figure S1).

**Figure 6:**
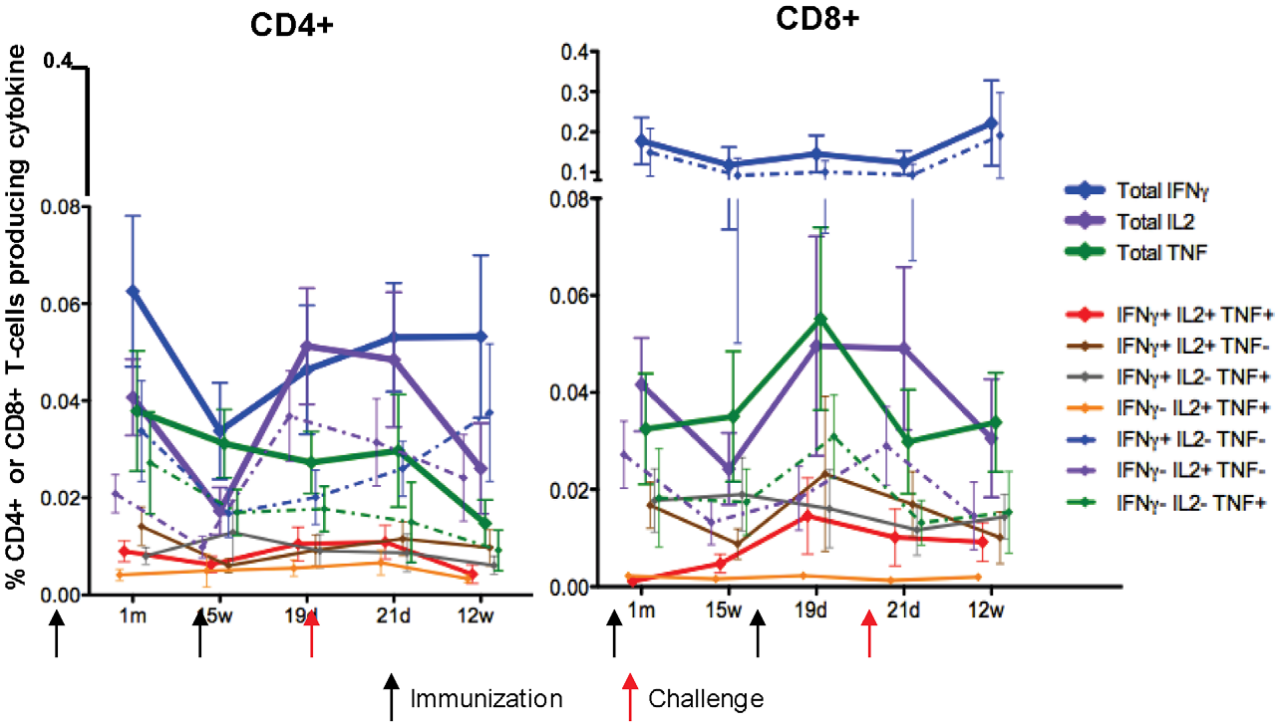
ICS CD4+ and CD8+ T cell multifunctional CSP responses. Arithmetic group mean percents with standard error of the mean for all cytokine phenotypes (total IFN-γ, total IL-2 or total TNF-α in bold solid lines, only IFN-γ, only IL2 or only TNF-α in dotted lines, and other subsets as non-bold solid lines). Responses prior to immunization are subtracted from all data in order to show only vaccine-induced responses.

All CD4+ and CD8+ T cell cytokine subsets were measured and the arithmetic means and standard deviations of each are shown in Figure 6. Overall, CD8+ T cells secreting only IFN-γ were the dominant phenotype across all categories. The different cytokine-secreting groups remained relatively constant as a proportion of total CD4 or CD8+ T cells during the course of the trial with no evidence of strongly changing ratios following the second immunization or immediately after challenge. An exception was CD4+ T cells secreting only IL2, a phenotype that rose after the second immunization, with total IL2 exceeding total IFN-γ at this time point. In addition, total CD8+ T cell IL2 and TNF-α also rose after the second immunization, but remained below total CD8+ T cell IFN-γ. Interestingly, measurements taken at a late time point – 12 weeks post challenge – showed that CD8+ and CD4+ T cell IFN-γ responses were sustained.

#### Antibody responses

**Figure 7:**
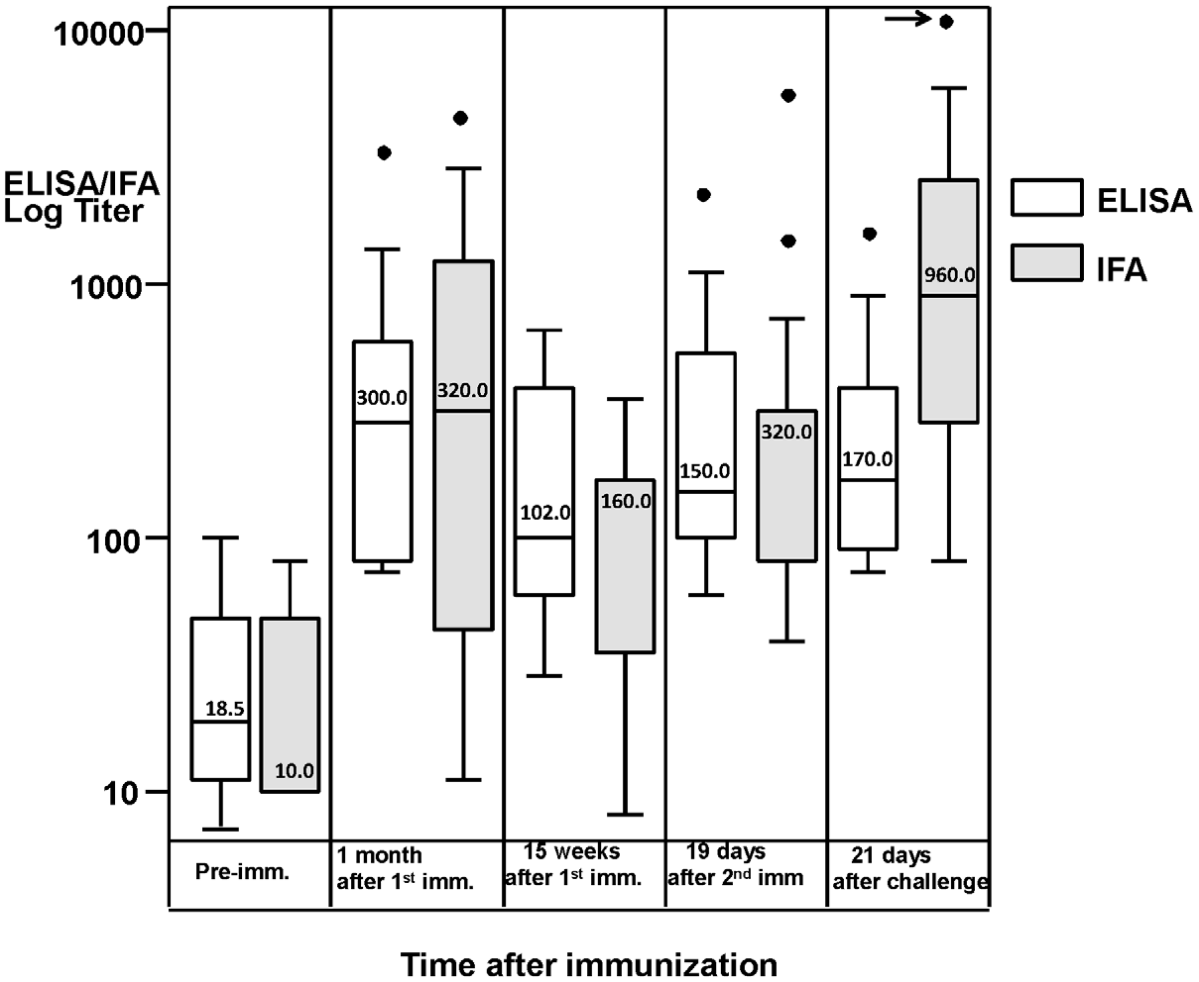
CSP ELISA and Sporozoite IFA activity. Box plots of the medians (50^th^ percentile) and 25^th^ and 75^th^ percentiles of ELISA and IFA activities prior to immunization, at 1 month and 15 weeks after the first immunization, 19 days after the second immunization, and 21 days after challenge. Whiskers spanning the first and third quartiles are at the base and top of each box, respectively, while the upper and lower horizontal bars at the ends of the whiskers represent the maximum and minimum values, respectively. Outliers are depicted by black dots; the arrow points to an outlier above the scale of the chart (1/20,480). The numbers give the value of the medians of each box, which in some cases are the same as the 25^th^ or 75^th^ percentile. When pre-challenge and post challenge titers were compared, ELISA remained similar (p = 0.15) but titers significantly increased for IFA (p = 0.02) using repeated analysis of log transformed values.

In general, anti-CSP antibody responses as measured by ELISA and sporozoite IFA were low following immunization, with the majority of titers recorded at less than 1∶1000 (Figure 7). Responses by ELISA peaked 4 weeks after the first immunization in six of 11 volunteers with exceptionally strong responses in one (v58), and at 15 weeks in one of 11 volunteers (v63), with negligible responses in four volunteers (v45, v52, v65 and v66). Patterns for IFA were similar. After the second immunization at week 16, titers rose in eight volunteers by each assay and showed little change for three volunteers; of the volunteers responding to the second immunization, only v63 significantly (by >2 fold) exceeded titers achieved after the first immunization. Thus, titers were similar for ELISA and IFA comparing after the first to after the second immunization (ELISA median 300 range 70–3579 after the first, and median 150 range 59–1980 after the second immunization; IFA median 320 range 10–2560 after the first, and median 320 range 40–5120 after the second immunization). Therefore, as with ELISpot, when the group was examined as a whole, there were no significant differences in humoral responses comparing the two immunizations (p = 0.42 for ELISA, 0.89 for IFA), even though there was a statistically significant increase in ELISA and IFA responses compared to pre-immunization titers with each immunization (p = 0.0001 for the first and 0.0398 for the second immunization for ELISA, and p = 0.0018 and 0.0177 for IFA). As with ELISpot, challenge had little effect on CSP ELISA titers in the group as a whole, while there was a statistically significant boosting of IFA titers (p = 0.02), indicating increased antibodies to the whole sporozoite, as would be expected following malaria infection.

#### Antigen interference: comparison of antibody and ELISpot responses to NMRC-MV-Ad-PfC and NMRC-M3V-Ad-PfCA

**Figure 8:**
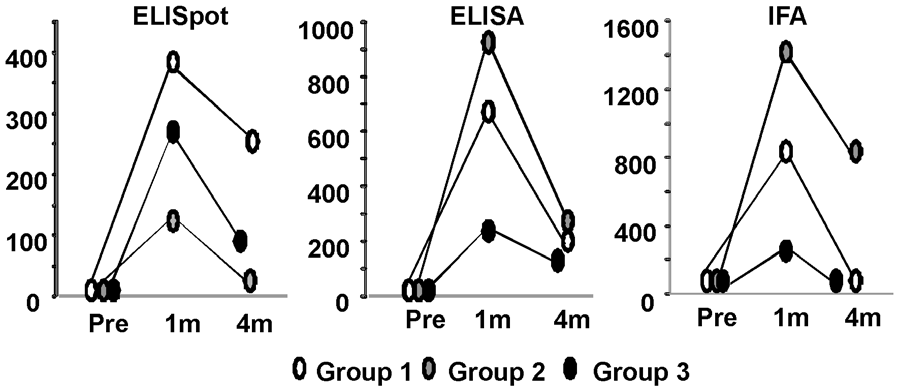
Comparing Groups 1, 2 and 3 for Immune Outcome Measures. The geometric means of ELISpot assay (CSP), ELISA (CSP) and IFA (sporozoite) in Groups 1, 2 and 3 are shown pre-immunization (Pre) and 1 and 4 months post immunization (1 month and 15 weeks post first immunization for Group 3). Groups 1 and 3 had better ELISpot responses than Group 2 (high dose), while Group 2 had the better ELISA and IFA responses (see Sedegah et al). Combination of the vector encoding CSP with the vector encoding AMA1 (Group 1) may have improved responses to CSP relative to the vector encoding CSP administered alone (Group 3), as immune responses were higher in Group 1 than in Group 3 for the three immune measures.

Group 3 offered the opportunity to look for interference between CSP and AMA1, since the CSP component of the vaccine, NMRC-MV-Ad-PfC, was administered in combination with the AMA1 component, NMRC-MV-Ad-PfA, to the volunteers in Group 1, and then was administered by itself, at the same dose, to volunteers in Group 3 (Figure 8). Combination with AMA1 in Group 1 did not reduce the immunogenicity of CSP by ELISpot (Group 1: geometric mean 422 sfc/million range 114–1066 sfc/m; Group 3: 323 sfc/million range 71–1127 sfc/m), ELISA (median Group 1 titer: 692 range 567–886; Group 3 titer: 300 range 70–3579), IFA (median Group 1 titer: 960 range 320–1280; Group 3 titer: 320 range 10–2560). Although not statistically significant, the trend was for improved responses when in combination, rather than diminished responses. This suggests that the adenovector backbone itself may have provided a degree of non-specific immune stimulation, since volunteers in Group 1 received twice the dose of adenovector as volunteers in Group 3 due to inclusion of the AMA1 component in Group 1.

#### Comparisons of low dose Groups 1 and 3 with high dose Group 2

For comparison purposes, Figure 8 includes ELISpot, ELISA and IFA responses to CSP for Group 2, which received a five-fold higher dose of NMRC-MV-Ad-PfC than Groups 1 or 3. ELISpot responses in Group 2 were significantly lower than in Group 1 (p = 0.0189) or Group 3 (p = 0.0009) when examined over the three time points, while ELISA and IFA responses in Group 2 trended higher than in Groups 1 or 3.

#### Correlations among immune responses

Immune responses were log_10_ transformed in order to conduct correlation analyses. CSP ELISA and sporozoite IFA responses were closely correlated with each other after the first immunization (correlation coefficient[r] = 0.826, p = 0.0017) and after the second immunization (r = 0.787, p = 0.004), as would be expected given that CSP is the dominant surface antigen of sporozoites. Neither antibody assay correlated with ELISpot responses (r<0.51, p>0.10 for each correlation), although interestingly all three of the volunteers whose ELISA titers never exceeded 100 (v045, v052, v066) were also low responders by ELISpot assay. The ELISA responses following the first and second immunizations were significantly correlated with each other (r = 0.666, p = 0.0252), and the same was true for IFA (r = 0.632, p = 0.0370) and for ELISpot (r = 0.738, p = 0.0095).

Although measuring different cell populations, ELISpot and ICS assays generally showed concordance. This was determined by comparing the dominant peptide pool as measured by ELISpot assay (Figure 4) and as measured by flow cytometry (Figure 5) for CD8+ T cells on day 28 after the first immunization. Discounting the low ELISpot responders (v045 and v068), in all other cases the dominant pool by ELISpot (or in the case of v063, one of the three equally dominant pools–see Figure 4) was also the dominant pool for CD8+ T cells by ICS assay (Figure 5), the only exception being v058 for which CD4+ T cell responses were stronger than CD8+ T cell responses and for whom the dominant pool for CD4+ T cells responses was concordant with the dominant pool for ELISpot responses.

### Efficacy

Twelve volunteers who completed both immunizations and were cleared from a safety perspective, plus 6 non-immunized infectivity controls, underwent malaria challenge by the bite of five *P. falciparum*-infected mosquitoes on day 21 following the second dose of vaccine. All 12 completed the challenge and follow-up, but one volunteer was removed from the analysis due revelation of previous participation in a malaria vaccine trial (information not disclosed at the time of enrollment). Therefore 11 volunteers were analyzed for immunogenicity and protection.

**Figure 9:**
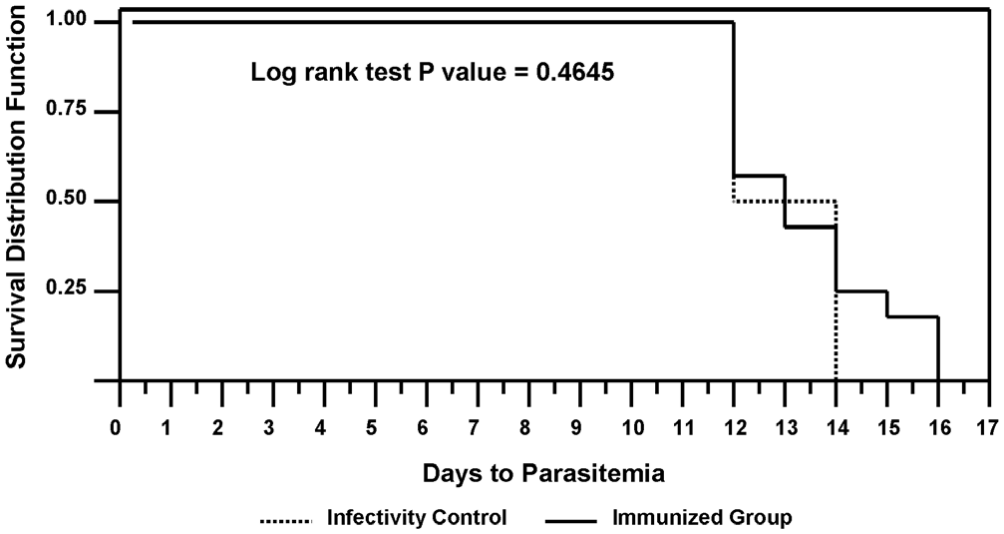
Vaccine Efficacy by Kaplan-Meier Plot. Two immunized volunteers became parasitemic on day 16, more than 2 standard deviations beyond the geometric mean time for the infectivity controls (day 13), indicating a significant delay. When days to patency were compared between the immunized and control groups, there was no difference observed (p = 0.46).

The challenge outcome is shown in Figure 9. There was no sterile protection as all volunteers developed parasitemia. The unimmunized infectivity controls became patent on days 12 (3/6) and 14 (3/6). The immunized volunteers became patent on days 12 (5/11), 13 (1/11), 14 (2/11), 15 (1/11) and 16 (2/11). There was no significant delay to parasitemia in the immunized group compared with the control group (Log Rank test: p = 0.46). The geometric means of time from infectious bite to patency were similar: immunized: 13.37 days, SD 1.63 days and controls: 12.96 days, SD 1.10 days. However, 2/11 immunized volunteers became patent on day 16, more than 2 standard deviations after the geometric mean of the time to patency for the controls (12.96+[2×1.10] = 15.1 days) indicating that these two volunteers (v45 and v69) had a delay in developing parasitemia. The NAb titers for v45 prior to the first and second immunizations were 82 and 924, respectively, and for v69 were <12 and 3600, respectively – thus one volunteer was considered seronegative at the time of first immunization (titer ≤12) while a second was considered low seropositive (>12 to 500).

### Pre-existing and vaccine-induced anti-Ad5 NAb responses

**Figure 10:**
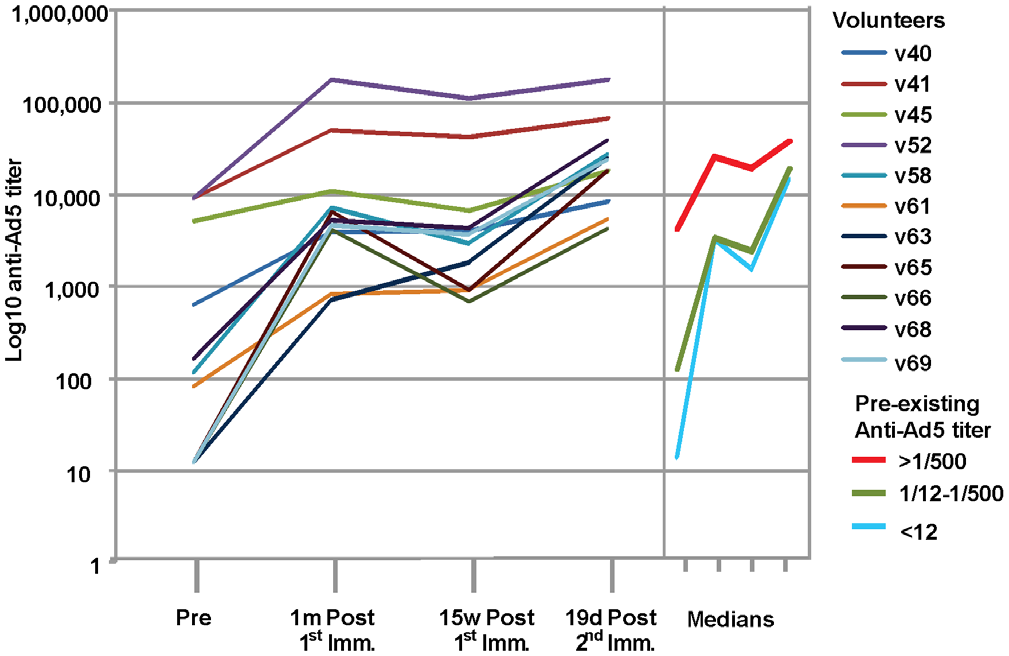
Anti-Ad5 NAb titers. Anti-Ad5 neutralization titers were measured pre-immunization (Pre) and 1 month and 15 weeks post 1^st^ immunization and 19 days post 2^nd^ immunization. Volunteers are shown in color-coded lines. Right Panel: median NAb titers of volunteers with high (>500), low (12–500) or absent (<12) titers at the time of enrolment.

For analysis, volunteers were divided into three groups: seronegative NAb>12; low levels NAb 12-500; and high levels NAb>500. Of the 11 volunteers assessed for immunogenicity and protection, four were Ad5 seronegative (v58, v61, v65 and v69), three had low levels of NAb (v41, v45, v68) and four had high levels (v40, v52, v63 and v66) at the time of screening (Table 3). The cut-off of 500 in the NVITAL assay is equivalent to the cut-off of 200 in the Merck assay [63]. The first dose of NMRC-MV-Ad-PfC induced NAb in volunteers who had been seronegative (median 4243, range 714-6317), and boosted pre-existing NAbs in volunteers who had low levels of NAb (median 5252, range 848–7285), and in volunteers with high levels of NAb (median, 30700, range 3893–177600) (Figure 10, right panel). After the second immunization, the majority of volunteers (nine of 11) developed responses that further exceeded activities after the first immunization (median titers seronegatives 20400 range 4142–23700, low seropositives 27900 range 5403–38100; and high seropositives 42300 range 8400–176100). The increases over time were highly significant for the seronegative and intermediate baseline groups (p<0.0001, p = 0.0026, respectively) but not for the initially high baseline group (p = 0.23).

### Effect of pre-existing and vaccine-induced anti-Ad 5 NAb on anti-CSP antibody and CMI responses

To examine the effect of NAb on immunogenicity, NAb titers prior to the first immunization were tested to determine if correlations existed with IFN-γ ELISpot counts, CD4+ T cells secreting IFN-γ by ICS, CD8+ T cells secreting IFN-γ by ICS, ELISA titers or IFA titers after the first and second immunizations (Table S2, section A). There were no significant correlations between pre-existing NAb titers and these immune measures. This was also true when correlations were performed between vaccine-induced NAb titers (measured just prior to the second immunization) and the same immune measures after the second immunization (Table S2, section B), although there was a non-significant trend for a negative effect on CD4+ T cells. The lack of an effect on immunogenicity (other than for CD4+ T cells) was particularly striking after the second immunization, as very high anti-Ad5 antibody activities were induced by the first immunization in some volunteers (Figure 10).

However, when fold changes in titers were correlated rather than the titers themselves, the fold-increase in NAb titer (calculated as titer prior to the second immunization divided by titer prior to the first immunization) showed a highly statistically significant negative correlation with the fold change in ELISA (calculated as titer following the second immunization divided by titer following the first immunization, a figure that in half the volunteers was less than 1 meaning that the second immunization did not further improve titers) (r = −0.76, p = 0.0062) (Table S2, section C). A marginally non-significant negative correlation was seen with IFA (r = −0.57, p = 0.069). However, no negative association was seen with ELISpot (r = +0.24, p = 0.48), nor with ICS CD4+ and CD8+ T cell IFN-γ responses, indicating that, like absolute titers of NAb, fold change in NAb had no influence on CMI responses in this trial.

**Figure 11:**
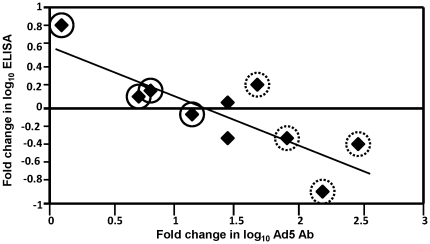
Negative Correlation between Fold Increase in Ad5 NAb Titer and Fold Change in CSP ELISA Titer. The ratio between Ad5 NAb titers induced by the first immunization (measured at 1 month) and titers prior to the first immunization (fold increase) was negatively correlated with the ratio between CSP ELISA titers induced by the second immunization (at 19 days) and titers induced by the first (at 1 month) (fold change). Volunteers with absent pre-existing immunity prior to the first immunization (titer <12, dotted circles) showed the greatest fold increase in NAb and, overall, the poorest boosting of antibody responses to CSP, while the volunteers with the highest pre-existing immunity (titer >500) (solid circles) showed the least fold increases in NAb and, overall, better boosting of antibody responses.

Interestingly, the four individuals with pre-existing titers <12 were the four with the greatest fold increase in NAb after the first immunization and three of the four showed the poorest antibody responses to the second dose of vaccine (Figure 11, points indicated with dashed circles). Conversely, the four volunteers with the highest pre-existing titers (>500) tended to show the least fold increases in NAb titers (Figure 11, points indicated with solid circles), and this was associated with improved induction of antibody responses following the second immunization. Overall, fold increase in NAb after the first immunization was negatively associated with fold change in antibody responses, comparing responses after the second immunization to responses after the first.

### Relationship between immunogenicity and efficacy

**Figure 12:**
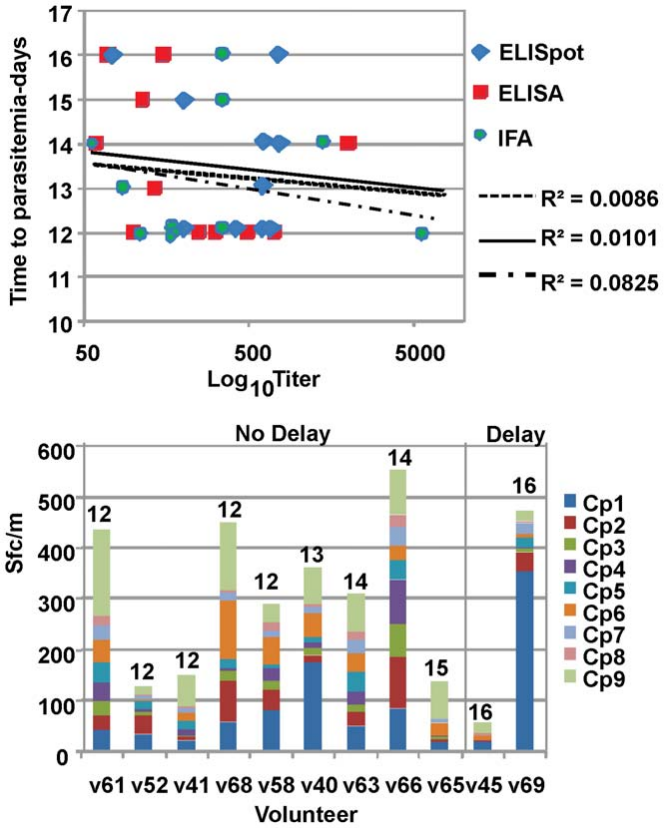
Relationship between pre-challenge ELISA (CSP), IFA (sporozoite) and ELISpot (CSP) activities and time to patency after challenge. **Upper Panel:** The time to patency in days after sporozoite challenge was compared to the pre-challenge magnitude of three immune measures: ELISA (CSP), IFA (sporozoite) and ELISpot (CSP). Best-fit trendlines are plotted with r^2^ values shown. There was no correlation between immune measures and time to patency. **Lower Panel:** Volunteers are arrayed according to time to patency (labeled in days at the top of each column), showing ELISpot activity to each color-coded CSP peptide pool. There is no evident relationship between magnitude of pool-specific or summed responses and time to patency.

When pre-challenge T cell responses by ELISpot and antibody titers by ELISA and IFA assays were compared to time to patency (Figure 12), there was no association between any of these measures and time to parasitemia ((p>0.1 for each predictor). The same was true for CD4+ and CD8+ T cell populations (p>0.1). When pre-challenge ELISpot responses to individual CSP peptide pools (Cp1–Cp9) were examined, one volunteer (v69) who became patent at 16 days was characterized by exceptionally strong ELISpot responses to one CSP peptide pool (Cp1) that were 2-fold greater than the next closest volunteer (v40), who did not have a significant delay of patency (Figure 12, lower panel). Therefore it is possible these T cell responses to Cp1 may have contributed to delayed patency in this volunteer. However, ELISpot responses in v45, who also had delayed parasitemia, were low. Thus there was no clear association between pool-specific responses and delay in parasitemia.

## Discussion

### Interpretation

#### Safety

The first objective of this study was to assess the safety and reactogenicity of the NMRC-MV-Ad-PfC vaccine. NMRC-MV-Ad-PfC (CSP component of parent vaccine NMRC-M3V-Ad-PfCA) given in two doses of 1×10^10^ pu was safe and well tolerated. The withdrawal of one volunteer after the first immunization was not vaccine-related. A second volunteer, with a history of migraine headaches, developed a migraine eight hours after the second immunization, possibly triggered by vaccine administration, and was excluded from participation in the malaria challenge. Pre-existing anti-Ad5 antibodies have been associated with decreased frequency of systemic AEs in other clinical trials [62], but no association could be determined comparing the reactogenicity profiles of seronegative and seropositive volunteers in this study.

When results from Group 3 and Groups 1 and 2 (Sedegah et al) were analyzed together the frequency of local adverse events were similar among the groups, However there were more systemic AEs in the high dose Group 2 volunteers (22 systemic AEs in six volunteers in six doses of vaccine; 3.7/vol/dose) than in low dose Group 1 volunteers (nine systemic AEs in six volunteers in six doses; 1.5/vol/dose) or low dose Group 3 volunteers (15 systemic AEs in 15 volunteers in 29 doses; 0.5/vol/dose). When pain or tenderness at the injection site, objective fever, myalgia, malaise and headache were examine separately from the other AE's, the AE rates between groups were statistically significantly different (Figure 2). An increasing incidence of adverse events related to dose has been recorded in other adenovectored vaccine trials, where a similar range of doses was administered [62], [64].

Neutropenia was the only apparent vaccine-related laboratory abnormality. Serotype 5 adenoviruses are thought to activate toll-like receptor 9 (TLR9) on dendritic cells [65]. When CpG, an immunostimulatory TLR9 agonist, was injected into human volunteers[66], [67] or injected with a malaria vaccine[68], it induced a transient neutropenia and injection site reactions that were linked to stimulation of the innate immune system and associated white blood cell sequestration[68]. Therefore, it is possible that the vaccine induced neutropenia through TLR9. As with CpG, neutropenia resolved within a week in most volunteers and did not appear to present any clinical risk.

#### Immunogenicity

The second objective of this study was to evaluate the immunogenicity of the NMRC-MV-Ad-PfC vaccine. In the first study of the parent vaccine (Sedegah et al), the lower vaccine dose (1×10^10^ pu) induced statistically significantly better ELISpot responses than the five-fold higher dose (5×10^10^ pu each construct), and for this reason the lower dose was selected for administration of NMRC-MV-Ad-PfC to Group 3. All volunteers developed increased ELISpot and antibody responses after the first immunization that generally peaked at 28 days, drifted down during the next 15 weeks and were boosted following the second immunization but generally did not exceed responses induced by the first immunization. IFN-γ responses to CSP as measured by ELISpot assay were in most cases robust, ranging in strength from 51 to 1128 sfc/m PBMCs, and were comparable to those induced by the RTS,S vaccine[69]. Although CD4+ T cell responses measured by ICS were lower than those reported in a recent RTS,S vaccine trial[69], RTS,S does not concurrently induce the robust CD8+ T cell responses recorded in this trial[70]. In contrast, antibody levels were low compared to those induced by RTS,S [15], [69], [71] and the kinetics of responses were different as at least two doses of RTS,S are generally required for maximum antibody responses [71], whereas a single dose of NMRC-MV-Ad-PfC induced peak responses in most volunteers.

Other clinical trials, in which two or more doses of adenovectored vaccines have been given, showed results consistent with this study. Repeat doses of adenovectored vaccines such as one encoding HIV *gag* (1×10^10^ pu dose) have been given at four weeks but have not significantly boosted responses over those induced by the first immunization [72], [73]. Likewise, a repeat dose of an adenovectored vaccine administered at three months did not improve responses to human cystic fibrosis [CF] transmembrane regulator relative to the first dose [74]. In that study, volunteers developed high titers of NAb after the first immunization, which was hypothesized to have affected the immunogenicity of the second dose.

Flow cytometry showed a predominance of CD8+ over CD4+ T cell IFN-γ responses for most (but not all) volunteers, as has been seen in other studies of adenovirus-vectored vaccines in humans[72] (Figure 5). IFN-γ single secretors were by far the most abundant phenotype among IFN-γ-secreting CD8+ T cells, indicating that the vaccine was a strong inducer of CD8+ effector T cells, but IFN-γ single secretors constituted only about half the population of IFN-γ-secreting CD4+ T cells (Figure 6). The T cell subpopulations secreting both IFN-γ and IL2 were negligible for both CD4+ and CD8+ T cells. The second immunization did not affect IFN-γ production as strongly as the first immunization; however, IFN-γ responses were remarkably sustained, especially for CD8+ T single secretors, which persisted strongly 12 weeks post challenge.

Proportions of T cells secreting IL-2 increased after the second immunization for both CD4+ and CD8+ T cells, to levels similar or higher that after the first, consistent with requirement for larger quantities of antigen to induce IL-2 compared with IFN-γ secretion. Thus a second dose of vaccine did not appear to be driving lymphocyte populations to a terminal single-secreting effector phase [73], [75]. On the contrary, the increase in IL2 secretion following the second immunization may reflect a strengthened reservoir of memory CD4+ T cells, and may have contributed to the sustained CD4+ T cell and CD8+ T cell IFN-γ responses mentioned above [75]. In the case of TNF-α, TNF-α-secreting CD4+ T cells appeared unaffected by the second immunization, whereas TNF-α-secreting CD8+ T cells increased along with the IL-2-secreting CD8+ cells. TNF-α-secreting CD8+ T cells fell during the ensuing weeks, whereas IL-2-secreting CD8+ cells were relatively sustained. The peaking of CD8+ T cell IL2 responses after CD8+ T cell TNF-α responses is consistent with a hierarchical control of CD8 + T cell cytokine responses seen in murine studies[75]. Triple-secreting T cells subpopulations occurred as a small percentage of both CD4+ and CD8+ T cells (Figure 6, and Supplementary Figure S1); interestingly, the two volunteers with delayed onset of parasitemia were among the three with the highest frequencies of multiply-positive CD8+ T cells. The evolution of different single and multifunctional CD4+ and CD8+ T cells subpopulations in response to NMRC-MV-Ad-PfC vaccine deserves further study and analysis.

#### Pre-existing and vaccine-induced Nab

Four of eleven volunteers evaluated for immunogenicity lacked any evidence of prior exposure to Ad5 (titers <12), while three had moderate titers (>12, <500) and four had high titers (>500). Contrary to expectations, naturally-acquired NAb had no appreciable effect on IFN-γ responses by ELISpot or ICS, or antibody responses by CSP ELISA or sporozoite IFA, as measured after the first or second doses of vaccine (Table S2, section A). Following the first immunization, however, the four seronegative volunteers experienced sharp increases in NAb titers compared to the seropositive volunteers (Figure 10). Although the resulting NAb titers again had no significant associations with measures of immunogenicity following the second immunization (Table S2, section B), three of the four originally seronegative individuals showed the poorest boosting of antibody responses to CSP following the second immunization among the eleven volunteers studied, while the originally seropositives individually boosted relatively well (Figure 10). In contrast with antibody responses, fold increase in NAb was not associated with positive or negative effects on ELISpot or ICS responses.

These differences likely reflect differential effects of naturally-acquired and vaccine-induced antibodies on the immune responses to the CSP transgene. A recent clinical study indicated that naturally-induced neutralizing antibodies were primarily directed to the Ad5 fiber whereas immunization with an adenovector induced neutralizing antibodies primarily directed to non-fiber capsid proteins (e.g., Ad5 hexon) [76]. The specificity of antibodies to various capsid proteins could variously affect trafficking and uptake of the vector by different cells types or tissues following vaccine administration, such that humoral but not cell-mediated responses were impacted. Antibodies to hexon protein, for example, could lead to viral uptake by APC's and differentially affect class I vs. class II presentation. It is possible that non-neutralizing anti-adenovirus antibodies may also modulate immune responses. As another possible mechanism, homing patterns of vaccine-induced T cells, could be affected by the type of pre-existing NAb, as was shown in a study in chimpanzees [77]. Various approaches to overcome the effects of neutralizing antibodies include replacing the hypervariable region of the adenovirus capsid with a malaria CSP sequence circumvented neutralization by pre-existing anti-adenovirus antibodies while maintaining immunogenicity[62]

Other studies suggest that the effect of pre-existing antibody on immune responses may be at least partially antigen dependent. In a study of an adenovectored HIV vaccine, ELISpot responses to gag but not to env appeared to be negatively affected by pre-existing immunity[62].

#### Interference

The immunogenicity of the CSP-encoding adenovector was similar in magnitude whether administered alone or mixed at the same dose with the AMA1-encoding adenovector (comparing the first dose in Group 3 with Group 1). Studies of mixtures of gene-based vaccines have shown that while many antigens may be compatible, some markedly inhibit the immunogenicity of other antigens[78], [79]. In this study, the combination of CSP with AMA1 had no negative impact on the immunogenicity of CSP. On the contrary, the double dose of adenovector in Group 1 compared with Group 3 may have provided a degree of non-specific immune stimulation since there was a non-significant trend toward improved immunogenicity in the mixture, consistent with findings that adenoviral vectors may activate innate and adaptive immune responses via the TLR9 or other receptors[79], [80], [81].

#### Efficacy

The third objective was to determine whether NMRC-MV-Ad-PfC provided protection against experimental *P. falciparum* sporozoite challenge. The CSP component was studied by itself in order to compare results with the protective RTS,S vaccine. No volunteer was protected, although two of 11 volunteers showed a delay to patency according to an accepted measure [71], [82]. Further evaluation of the immune responses elicited by these two volunteers revealed high levels of IFN-γ to CSP peptide pool Cp1 in one of these volunteers (two fold higher than in any other volunteer), but low responses in the other, precluding any clear association between these responses and delay. Interestingly, these two volunteers were among the three volunteers showing the highest frequencies of multifunctional CD8+ T cells.

Adenovirus-vectored *P. yoelii* CSP induced sterile immunity in 40% of immunized mice and reduced liver stage parasite load in the remaining mice by up to 93% resulting a prolonged pre-patent period [53]. Therefore, the partial protection seen in this clinical trial could be interpreted as consistent with the murine study, since the delay to parasitemia in two volunteers may also have resulted from a reduction in the numbers of liver stages by vaccine-induced CD4+ and/or CD8+ T cells.

The results of this study contrast with the RTS,S vaccine, also based on CSP, that sterilely protects roughly 41% of malaria-naïve volunteers against experimental sporozoite challenge when adjuvanted with AS02 [71]. RTS,S induces high antibody responses and CD4+ T cell responses that have been correlated with protection[69]. In this study, the magnitudes of antibody and CD4+ T cell responses elicited by the NMRC-MV-Ad-PfC vaccine were likely insufficient to reproduce the protective mechanism of RTS,S. Even though robust CD8+ T cell responses were induced, they may likewise have been insufficient to induce protection via cell-mediated cytotoxicity. Prolonged antigen loads may affect CD8+ T cell responses and perhaps, therefore, is a consequence of two immunizations with the NMRC-MV-Ad-PfC vaccine[83], [84], suggesting that the quality rather than quantity of CD8+ T cell may be as important in mediating protection. Therefore, it would be useful to test heterologous prime-boost regimens able to induce higher levels of CMI, and to include additional pre-erythrocytic stage antigens in the vaccine to see if the adenovirus platform is more suitable for antigens other than CSP.

### Generalizability

The favorable tolerability and safety profile of the NMRC-MV-Ad-PfC vaccine is consistent with other studies of Ad5 vaccines encoding non-malarial antigens administered at similar doses. Likewise, the immunogenicity recorded in this trial is concordant with other trials of Ad5-vectored vaccines.

The absence of sterile protection following immunization with NMRC-MV-Ad-PfC may be related to the antigen studied or to the delivery system. Other malaria antigens, particularly other liver stage antigens, should be tested, as well as prime-boost approaches. These strategies may augment T cell and antibody responses, and may also improve the quality of the T cell response to protect. For example, prime-boost approaches have been shown to increase the magnitude of both antibody and T cell responses and may induce different epitope specificities, or different cytokine profiles in the responding T cells.

### Limitations

The small sample size of this first-in-humans study limits the power to draw firm conclusions regarding the impact of NAb on the immunogenicity of the vaccine: seven volunteers had titers <500, and four volunteers had titers >500. Nevertheless, results from these 11 volunteers demonstrated a divergent effect of NAb on humoral and cell-mediated responses. The data indicated that an absence of pre-existing NAb led to stronger vaccine-induced NAb which may have subsequently blunted the humoral response to a second dose of vaccine, whereas ELISpot responses were unaffected. However, these results need confirmation. In particular, more data are needed to differentiate the effects of naturally-acquired vs. vaccine-induced NAb, and to document whether or not the effects of pre-existing immunity are antigen-dependent.

### Overall evidence

This trial confirms other studies demonstrating the safety and tolerability of adenovector vaccines in the 1×10^10^−1×10^11^ pu dose range, provides additional evidence of the potency of adenovectors for inducing CD8+ IFN-γ responses targeting malaria antigens in humans, suggests that a second dose does not enhance the immunogenicity of a first dose, and that CSP does not offer significant protection when delivered by itself using an Ad5 platform. Improved efficacy against *P. falciparum* may be achieved by employing heterologous prime-boost strategies or by adding other malaria antigens to the vaccine.

## Supporting Information

Figure S1 **ICS CD4+ and CD8+ multicytokine T cell responses (CSP).** Panels A & C: Multifunctional (any two or more cytokines among IFN-γ, IL2 or TNF-α) CD4+ and CD8+ T cell activity of each volunteer in response to CSP peptides at (1) 1 month and (2) 15 weeks after the first immunization, (3) 19 days after the second immunization, and (4) 21 days after challenge, as stacked, color-coded peptide pool-specific responses at each time point. Responses prior to immunization were subtracted in order to show only vaccine-induced responses. Panels B & D: Pie charts representing the proportion of secreting cells that were single cytokine secretors, double cytokine secretors or triple cytokine secretors; numbers on pie charts represent percents. Panel E: The values of the sum of pool-specific responses for each volunteer at each time point listed in Panels A and C plus and additional time point 12 weeks post challenge. The horizontal bar indicates the geometric mean of the group.
(TIF)

Table S1 **Unsolicited adverse events definitely, probably or possibly related to immunization.** Unsolicited adverse events were recorded for 28 days following each immunization.
(DOC)

Table S2 **Rank correlations between pre-existing and vaccine-induced anti-Ad5 NAb titers and CSP ELISpot, CD4+ T cell and CD8+ T cell IFN-γ activities and CSP ELISA and Sporozoite IFA titers post 1^st^ and 2^nd^ immunizations.** A: Pre-existing Ad5 NAb titers measured prior to the first immunization were tested to see if they were correlated with CSP IFN-γ ELISpot, total IFN-γ CD4+ T cells by ICS, total IFN-γ CD8+ T cells by ICS, CSP ELISA and sporozoite IFA activities for 11 volunteers in Group 3 for both the first and second immunizations. The upper number in each paired entry is the rank correlation coefficient (r) and the lower number is the p-value for the null hypothesis that the correlation is zero. No significant correlations were identified. B: Vaccine-induced Ad5 NAb titers measured prior to the second immunization were correlated with the same immune measures after the second immunization. Again, no significant correlations were identified, although there was a trend toward a negative effect on CD4+ T cells (p = 0.089). C: The fold-increases in Ad5 NAb titers from before the first to before the second immunization were calculated, and correlations were computed. There were no significant correlations identified with the same immune measures after the first or second immunizations, but when fold changes were calculated in these immune measures (fold changes from activities one month following the first immunization to activities 19 days following the second immunization), a highly significant negative correlation was identified for ELISA (bold) and a non-significant trend for IFA (p = 0.0686). This correlation for ELISA is shown graphically in Figure 7. In contrast, no association was evident for ELISpot responses, CD4+ T cell responses or CD8+ T cell responses, for which correlation coefficients were positive and non-significant. Rank correlation coefficients and p-values were obtained from SAS.
(DOC)

Protocol S1 (DOC)

Checklist S1 (DOC)
