# Supplementary figures and images for: Adenovirus-5-Vectored P. falciparum Vaccine Expressing CSP and AMA1. Part B: Safety, Immunogenicity and Protective Efficacy of the CSP Component
Source: PLoS One. 2011 Oct 7;6(10):e25868. doi: 10.1371/journal.pone.0025868 (PMC3189219; doi:10.1371/journal.pone.0025868)

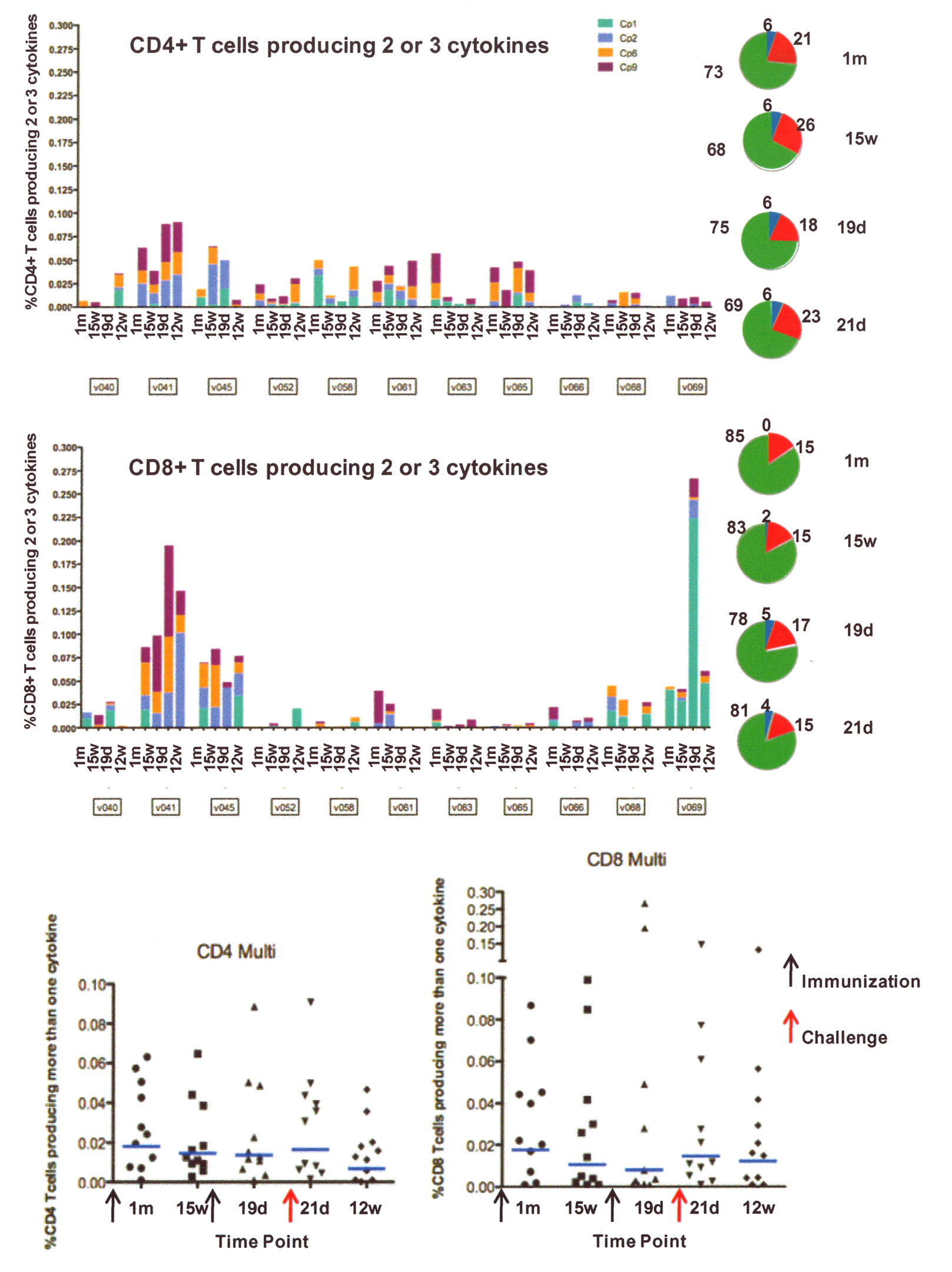

Supplement: Figure S1 — ICS CD4+ and CD8+ multicytokine T cell responses (CSP). Panels A & C: Multifunctional (any two or more cytokines among IFN-γ, IL2 or TNF-α) CD4+ and CD8+ T cell activity of each volunteer in response to CSP peptides at (1) 1 month and (2) 15 weeks after the first immunization, (3) 19 days after the second immunization, and (4) 21 days after challenge, as stacked, color-coded peptide pool-specific responses at each time point. Responses prior to immunization were subtracted in order to show only vaccine-induced responses. Panels B & D: Pie charts representing the proportion of secreting cells that were single cytokine secretors, double cytokine secretors or triple cytokine secretors; numbers on pie charts represent percents. Panel E: The values of the sum of pool-specific responses for each volunteer at each time point listed in Panels A and C plus and additional time point 12 weeks post challenge. The horizontal bar indicates the geometric mean of the group. (TIF) [file pone.0025868.s001.tif]
