# Supplementary material for: Adenovirus-5-Vectored P. falciparum Vaccine Expressing CSP and AMA1. Part B: Safety, Immunogenicity and Protective Efficacy of the CSP Component
Source: PLoS One. 2011 Oct 7;6(10):e25868. doi: 10.1371/journal.pone.0025868 (PMC3189219; doi:10.1371/journal.pone.0025868)
Supplement: Table S1 — Unsolicited adverse events definitely, probably or possibly related to immunization. Unsolicited adverse events were recorded for 28 days following each immunization. (DOC) [file pone.0025868.s002.doc]

| **Adverse event** | **Vol #** | **Day** | **Grade** | **Relatedness** | **Comments** |
| --- | --- | --- | --- | --- | --- |
| Numbness at injection site | V45 | 0 | 1 | Definite | 2-3 cm area at injection site, occurred immediately post injection, resolved within 2 hours |
| Burning at injection site | V58 | 0 | 1 | Definite | Occurred immediately post injection, resolved by the next day |
| Bruise at injection site | V58 | 1 | 1 | Definite | Resolved by day 6 post immunization |
| Bruise at injection site | V63 | 2 | 1 | Definite | 3.5 by 2.5 cm bruise at injection site, resolved by day 11 |
| URI | V58 | 18 | 1 | Possible | Sore throat with nasal congestion days 18-28 post immunization |
|  |  |  |  |  |  |
| Dry itchy eyes | V65 | 4 | 1 | Possible | Intermittent episodes of dry, itchy eyes days 4 - 11, without conjunctivitis or exudates on examination; nasal congestion with headache day 13. |
| Photo & sound sensitivity | V48 | 1 | 1 | Possible | Typical migraine headache 8-10 hours following immunization, associated with 3 episodes of chills. |
