# Supplementary material for: Adenovirus-5-Vectored P. falciparum Vaccine Expressing CSP and AMA1. Part B: Safety, Immunogenicity and Protective Efficacy of the CSP Component
Source: PLoS One. 2011 Oct 7;6(10):e25868. doi: 10.1371/journal.pone.0025868 (PMC3189219; doi:10.1371/journal.pone.0025868)
Supplement: Table S2 — Rank correlations between pre-existing and vaccine-induced anti-Ad5 NAb titers and CSP ELISpot, CD4+ T cell and CD8+ T cell IFN-γ activities and CSP ELISA and Sporozoite IFA titers post 1st and 2nd immunizations. A: Pre-existing Ad5 NAb titers measured prior to the first immunization were tested to see if they were correlated with CSP IFN-γ ELISpot, total IFN-γ CD4+ T cells by ICS, total IFN-γ CD8+ T cells by ICS, CSP ELISA and sporozoite IFA activities for 11 volunteers in Group 3 for both the first and second immunizations. The upper number in each paired entry is the rank correlation coefficient (r) and the lower number is the p-value for the null hypothesis that the correlation is zero. No significant correlations were identified. B: Vaccine-induced Ad5 NAb titers measured prior to the second immunization were correlated with the same immune measures after the second immunization. Again, no significant correlations were identified, although there was a trend toward a negative effect on CD4+ T cells (p = 0.089). C: The fold-increases in Ad5 NAb titers from before the first to before the second immunization were calculated, and correlations were computed. There were no significant correlations identified with the same immune measures after the first or second immunizations, but when fold changes were calculated in these immune measures (fold changes from activities one month following the first immunization to activities 19 days following the second immunization), a highly significant negative correlation was identified for ELISA (bold) and a non-significant trend for IFA (p = 0.0686). This correlation for ELISA is shown graphically in Figure 7. In contrast, no association was evident for ELISpot responses, CD4+ T cell responses or CD8+ T cell responses, for which correlation coefficients were positive and non-significant. Rank correlation coefficients and p-values were obtained from SAS. (DOC) [file pone.0025868.s003.doc]

|  | Ad5 Nab | Time Point | Immune Measure Correlations (r, p value) | | | | |
| --- | --- | --- | --- | --- | --- | --- | --- |
|  |  |  | ELISpot | CD4 | CD8 | ELISA | IFA |
| A | Pre 1^st^ immun  (pre-existing) | Post 1^st^ immun | -0.1679  0.6217 | -0.4850  0.1305 | -0.2658  0.4295 | -0.3871  0.2396 | -0.4568  0.1578 |
|  |  |  |  |  |  |  |  |
|  |  | Post 2^nd^ immun | 0.0233  0.9457 | -0.4430  0.1723 | -0.2145  0.5265 | -0.2239  0.5082 | -0.0760  0.8242 |
|  |  |  |  |  |  |  |  |
| B | Pre 2^nd^ immun  (vaccine-induced) | Post 2^nd^ immun | 0.2455  0.4669 | -0.5364  0.0890 | -0.0909  0.7904 | -0.1000  0.7699 | 0.0191  0.9555 |
|  |  |  |  |  |  |  |  |
| C | Fold change  Pre 1^st^ to Pre 2^nd^ immun | Post 1^st^ immun | 0.0364  0.9155 | 0.1727  0.6115 | -0.0182  0.9577 | 0.4364  0.1797 | 0.4932  0.1232 |
|  |  |  |  |  |  |  |  |
|  |  | Post 2^nd^ immun | 0.2909  0.3855 | 0.1546  0.6500 | 0.3000  0.3701 | 0.1455  0.6696 | 0.0430  0.9001 |
|  |  |  |  |  |  |  |  |
|  |  | Fold change  Post 1^st^ to Post 2^nd^ | 0.2364  0.4841 | 0.1909  0.5739 | 0.2455  0.4669 | **-0.7636**  **0.0062** | -0.5676  0.0686 |
